# Safety and Efficacy of Vaccines During Pregnancy: A Systematic Review

**DOI:** 10.7759/cureus.77176

**Published:** 2025-01-09

**Authors:** Lakshmi Venkata Sharmista Chittajallu, Rohini Kaku, Poshitha Kondadasula, Jun Yi Lim, Altynai Zhumabekova

**Affiliations:** 1 Department of Obstetrics and Gynecology, Command Hospital (Central Command), Lucknow, IND; 2 Department of General Medicine, I.K. Akhunbaev Kyrgyz State Medical Academy, Bishkek, KGZ; 3 Department of General Medicine, A. C. Subba Reddy Government Medical College, Nellore, IND; 4 Department of Obstetrics and Gynecology, City Maternity Hospital No. 2, Bishkek, KGZ

**Keywords:** antibody transfer, coronavirus of 2019, diphtheria tetanus pertussis, efficacy, immunogenicity, influenza, maternal vaccination, neonatal outcomes, pregnancy, respiratory syncytial virus

## Abstract

Maternal immunization is a safe and effective strategy for protecting mothers and infants from vaccine-preventable diseases. This systematic review evaluated the safety and efficacy of various vaccines administered during pregnancy, focusing on maternal and infant outcomes. A comprehensive literature search was conducted in PubMed, Scopus, and Web of Science to identify relevant studies. The search used terms and combinations such as (“maternal vaccination” OR “vaccination during pregnancy”) AND (“safety” OR “efficacy” OR “immunogenicity”) AND (“influenza” OR “DTaP” OR “respiratory syncytial virus” OR “group B streptococcus” OR “COVID-19”). Boolean operators “AND” and “OR” enhanced precision and filtered the limited results to studies published from 2018 to 2024. Eight studies were included in the review after applying inclusion and exclusion criteria. Influenza, diphtheria-tetanus-pertussis, respiratory syncytial virus, group B streptococcus, and COVID-19 vaccines are safe and effective when administered during pregnancy. These vaccines elicit robust immune responses in pregnant women, with efficient transplacental antibody transfer providing passive immunity to newborns. Adverse effects were mostly mild to moderate and similar to those observed in nonpregnant individuals. No significant increase in adverse pregnancy or neonatal outcomes was associated with maternal vaccination. Most of the included randomized controlled trials (had a low risk of bias, thus supporting the reliability of the findings. However, vaccine hesitancy remains a challenge, highlighting the need for transparent communication between healthcare providers and pregnant women. Future research should focus on long-term infant health outcomes, vaccine safety, immunogenicity in diverse populations, and strategies to optimize maternal immunization timing and enhance neonatal antibody persistence. This review supports the implementation of routine maternal vaccination programs and emphasizes the importance of addressing knowledge gaps and ensuring equitable access to immunization during pregnancy.

## Introduction and background

Vaccination during pregnancy protects both mother and infant, particularly during the newborn period. Passive antibody transfer through the placenta offers immediate protection to newborns before routine vaccination [1]. Maternal diphtheria-tetanus-pertussis (DTaP) vaccination significantly lowers the risk of pertussis, which can cause severe complications and death in infants [2]. Prenatal influenza vaccination is associated with reduced flu-related hospitalizations in infants up to six months of age [3]. These findings highlight the dual benefit of maternal immunization: protecting mothers and newborns during high-risk infection periods.

Research indicates that mRNA COVID-19 vaccines administered during early pregnancy do not increase the risk of major structural birth defects in live-born infants [4]. More extensive cohort studies have found no association between prenatal mRNA COVID-19 vaccination and high risks of adverse pregnancy outcomes, such as preterm birth, small-for-gestational-age infants, gestational diabetes, or hypertensive disorders [5].

Studies on other vaccines, such as the non-adjuvanted bivalent respiratory syncytial virus (RSV) vaccine, have also confirmed their safety during pregnancy, with no increased risk of preterm birth [6]. Studies on seasonal influenza vaccination across multiple pregnancies have shown no heightened risk of adverse perinatal outcomes, supporting the recommendations for influenza vaccination during pregnancy [7].

Despite these positive findings, vaccine hesitancy persists because of concerns about the long-term effects and unclear information on vaccine safety and efficacy during pregnancy. Transparent communication between healthcare providers and public health organizations is essential to address these concerns [8].

Ongoing research on vaccine safety and effectiveness during pregnancy is crucial to address expectant mothers’ concerns and provide evidence-based recommendations. A recent meta-analysis from various countries has shown that COVID-19 vaccination during pregnancy significantly reduced the risk of severe disease in mothers and provided measurable protection to infants, as indicated by cord blood antibody levels [9]. Despite these benefits, ongoing concerns regarding vaccine safety require more long-term studies and comprehensive communication strategies to address the benefits and uncertainties of prenatal vaccination [10]. Collaborative efforts between healthcare providers and public health organizations can bridge these gaps and promote informed decisions that safeguard maternal and infant health.

This review evaluates the safety and effectiveness of various vaccines administered during pregnancy, considering their effects on mothers and infants. It focuses on the immune responses, side effects, and protective benefits for newborns. By thoroughly analyzing the advantages and potential risks of maternal vaccination, this review aims to alleviate concerns about vaccine safety and efficacy in pregnant women, ultimately contributing to evidence-based guidelines for maternal vaccination.

## Review

Materials and methods

This review followed the Preferred Reporting Items for Systematic Reviews and Meta-Analyses (PRISMA) guidelines [11]. We conducted a systematic review to assess the safety and efficacy of various vaccines administered during pregnancy, focusing on maternal and infant outcomes.

A comprehensive literature search was conducted in PubMed, Scopus, and Web of Science to identify relevant studies. The search used terms and combinations such as (“maternal vaccination” OR “vaccination during pregnancy”) AND (“safety” OR “efficacy” OR “immunogenicity”) AND (“influenza” OR “DTaP” OR “respiratory syncytial virus” OR “group B streptococcus” OR “COVID-19”). Boolean operators “AND” and “OR” enhanced precision and filtered limited results to studies published from 2018 to 2024.

The search focused on peer-reviewed publications with full-text availability, publication in the English language, published between 2018 and 2024 to ensure the availability of updated information. The inclusion criteria were vaccines administered during pregnancy, reported maternal and neonatal safety outcomes, immunogenicity, efficacy, randomized controlled trials (RCTs), cohort studies, and case-control studies. The exclusion criteria were studies not centered on vaccines administered during pregnancy, review articles, case reports, letters to the editor, or animal studies were excluded. Furthermore, papers without accessible full text or outcome data were excluded.

Key findings from each study are presented, emphasizing adverse events, antibody transfer rates, and infant health indicators. The synthesis focused on the immune response in both mothers and newborns and any adverse pregnancy or neonatal outcomes linked to maternal vaccination.

Data were extracted using a standardized form by two independent reviewers who focused on the authors, publication year, study design, sample size, duration, age and sex distribution, baseline attributes, and inclusion and exclusion criteria. Disagreements during data extraction were resolved through discussion, and a third reviewer was consulted if necessary. Ethical considerations were based on the reported ethical approval of the included studies; no additional ethical approval was required as the review used publicly available data.

A systematic evaluation of the potential bias in the included studies was conducted using established assessment tools tailored to each study design. For RCTs, the Cochrane Risk of Bias Tool was used to examine various bias types, including selection, performance, detection, reporting, and other potential biases, such as those related to funding. Each category was assigned a low-, moderate-, or high-risk level. The ROBINS-I Tool was used for non-RCTs, focusing on confounding factors, participant selection, intervention classification, deviations from intended interventions, missing data, outcome measurements, and reporting bias. The assessment process involved two independent reviewers, and any disagreements were resolved through discussion or consultation with a third reviewer. This thorough approach ensured a transparent and reliable evaluation of the study.

Results

**Figure 1:**
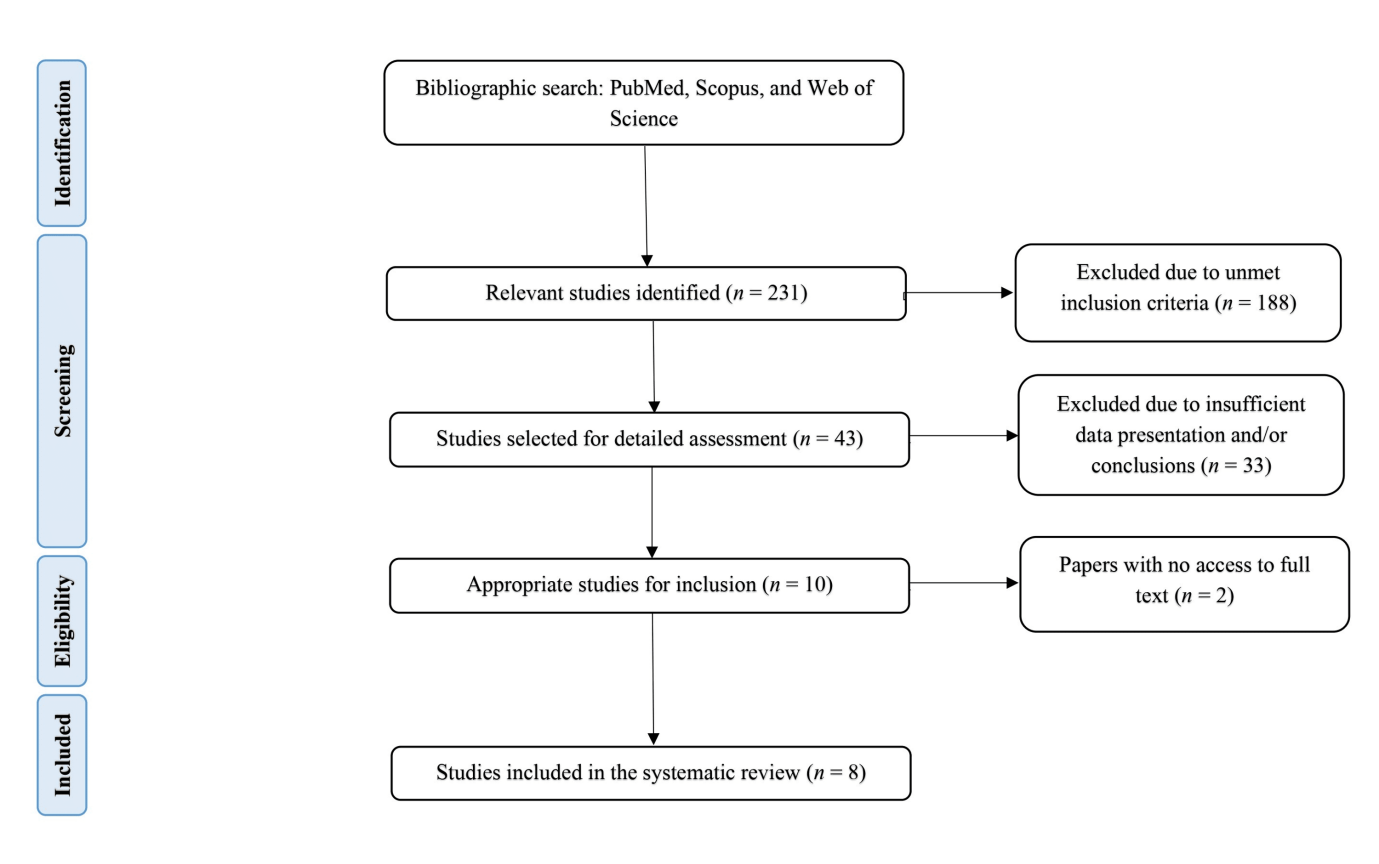
Flow diagram of literature search and study of selection for systematic review (PRISMA flow chart) PRISMA: Preferred Reporting Items for Systematic Reviews and Meta-Analyses

The initial literature search yielded 231 results, which were filtered according to specific inclusion and exclusion criteria. Of the 231 articles, 188 were excluded because they did not meet the inclusion criteria owing to inappropriate research designs (e.g., preclinical or pediatric studies), insufficient reporting of results, or lack of quantitative data on vaccines during pregnancy, safety, and efficacy. Some studies were inaccessible, despite institutional access requests and direct author communication. The remaining 43 articles were subjected to comprehensive analysis, of which 33 were excluded if they lacked relevant quantitative outcome data, detailed methodology or findings, or statistically supported conclusions. Of the 10 remaining articles, two were inaccessible in full text despite multiple retrieval attempts, leaving eight studies for systematic review [12-19]. Figure 1 illustrates the selection process, and Table 1 lists the eight studies selected for this systematic review.

**Table 1:** Characteristics of selected studies on the safety and efficacy of vaccinations administered during pregnancy DTaP: diphtheria tetanus pertussis; GBS: group B streptococcus; IIV3: trivalent inactivated influenza vaccine; IIV4: quadrivalent inactivated influenza vaccine; RSV: respiratory syncytial virus; Td: tetanus-diphtheria

Study details	Study objectives	Study design	Intervention	Main findings
Kostinov et al. [12]	This study evaluated the safety and immune response of pregnant women to influenza vaccines, both adjuvant and non-adjuvant using subunit formulations.	Prospective, randomized, open-label, comparative, parallel-group trial in pregnant women, with a placebo control group	Thirty-seven pregnant women received a single dose of the non-adjuvanted Agrippal S1 vaccine between weeks 16 and 30 of pregnancy. Forty-two expectant mothers were administered a single dose of adjuvanted Grippol Plus vaccine between the 16th and 30th weeks of gestation.	Pregnant women tolerated both non-adjuvant and adjuvant subunit influenza vaccines well, with local reactions reported in <10% of recipients. The non-adjuvant vaccine-induced protective antibodies in 64.8-94.5% of individuals, while the adjuvant vaccine achieved this in 72.5-90.0%. After nine months, antibody levels persisted at 51.3-72.9% in the non-adjuvant group and 54.2-74.2% in the adjuvant group, indicating ongoing protection.
Munoz et al. [13]	This study evaluated the effectiveness, immune response, and duration of maternal antibody transfer of three licensed seasonal IIV3s administered to pregnant women.	Prospective, randomized, double-blind, parallel-group study with a control group of nonpregnant women	A single intramuscular injection of an authorized seasonal IIV3: Agriflu^®^, Fluzone^®^, or Fluarix.	Pregnant and nonpregnant women tolerated the IIV3 vaccine well without significant adverse reactions. Antibody responses were similar in both groups. Maternal antibodies were transferred to infants but declined quickly within the first six weeks postpartum.
Vesikari et al. [14]	This study compared immune responses to IIV4 and IIV3 in pregnant women 21 days after vaccination, evaluated the safety of IIV4 versus IIV3 in these women, examined placental antibody transfer to infants, and assessed the safety profiles and birth outcomes.	A randomized, double-blind, controlled, multicenter study	A single dose of 0.5 ml intramuscular injection of either IIV4 or IIV3 was administered.	Both IIV4 and IIV3 similarly increased HAI antibody titers for the common influenza A and B strains, but IIV4 produced higher titers for the additional B/Yamagata strain. Vaccination of pregnant women with IIV4 or IIV3 leads to a significant transplacental transfer of influenza antibodies to newborns. The safety profiles of IIV4 and IIV3 were similar, with comparable rates of mild solicited and unsolicited adverse reactions.
Munoz et al. [15]	To assess the safety and immunogenicity of the two approved seasonal IIV3 in pregnant women	A prospective, randomized, controlled clinical trial	A single intramuscular injection of either Fluzone^®^ or Fluarix^®^ that was approved for seasonal IIV3.	Pregnant women typically tolerate seasonal flu doses well, with mild side effects and no serious adverse reactions. The vaccines effectively stimulated immune responses in expectant mothers, with strong antibody production against influenza A variants but weaker responses to influenza B strains. Overall, seasonal influenza immunization was deemed safe and effective during pregnancy.
Halperin et al. [16]	This study assessed the safety of administering the Tdap vaccine during pregnancy and the immune responses elicited in pregnant women and evaluated the impact of maternal Tdap immunization on infants’ immune responses to vaccines administered at two, four, and six months as well as the booster shot at 12 months.	Randomized, controlled, observer-blind, parallel-group, multicenter clinical trial	Tdap and Td vaccine during pregnancy	The safety of the Tdap vaccination during pregnancy mirrored that of the Td vaccination. Newborns of Tdap-vaccinated mothers exhibited higher pertussis antibody levels at birth than did their mothers. These infants maintained elevated antibody levels early in life, but during the later stages of the primary vaccination series, their levels were lower than those whose mothers received Td.
Madhi et al. [17]	This study evaluated the safety and immunogenicity of RSV F-protein nanoparticle vaccine administration in pregnant women and determined its efficacy in preventing RSV-related medically significant lower respiratory tract infections in infants.	A multi-center randomized, double-blind, placebo-controlled trial	A nanoparticle RSV F-protein vaccine or placebo was administered intramuscularly to pregnant women, with the vaccine group 2:1 of the control group.	When administered to pregnant women, the RSV F-protein vaccine proved safe and immunogenic, eliciting antibodies that were transferred to their infants. It was 39.4% effective in reducing RSV-specific significantly lower respiratory tract infections in infants up to 90 days of age and showed 58.8% efficacy in decreasing RSV lower respiratory tract infections with severe hypoxemia in infants within the same age range.
Swamy et al. [18]	This study assessed the safety and immune response of a new trivalent GBS vaccine that was administered to pregnant women. It also investigated the placental transfer of serotype-specific antibodies, their persistence in infants, and their presence in the breast milk of vaccinated mothers.	A randomized, observer-blind, placebo-controlled trial	Single administration of trivalent GBS vaccination to pregnant women at 24-34 weeks of gestation	Pregnant women receiving the trivalent GBS vaccine showed a good safety profile and elevated antibody production, which was passed on to their infants and remained detectable for at least three months. Infants of vaccinated mothers had higher antibody levels in both the blood and breast milk than those of placebo recipients.
Yang et al. [19]	This study investigated the safety of inactivated COVID-19 vaccines during peripregnancy, assessing maternal premature rupture of membranes and adverse neonatal outcomes, such as induced labor or mortality, preterm delivery, low birth weight, and neonatal intensive care unit admission. The study also examined various secondary outcomes in both mothers and their newborns.	Observational cohort studies using a parallel group design assigned participants to vaccine or control groups based on vaccination status rather than randomization	Inactivated COVID-19 vaccine administered during the peri-pregnancy period	The inactivated COVID-19 vaccine was safe for pregnant women and their offspring, irrespective of vaccination timing or medication regimen, with no significant differences in premature membrane rupture or adverse neonatal outcomes between the vaccinated and control groups. However, the vaccinated group showed higher serum alanine transaminase levels during the first trimester.

Maternal vaccination has been shown to be safe and effective in protecting expectant mothers and their infants from various infectious diseases. Influenza vaccines have demonstrated good tolerability and robust immune responses in pregnant women, leading to effective newborn antibody transmission [12-15]. The DTaP vaccine increased pertussis antibody levels in neonates but reduced levels after initial immunization [16]. Maternal RSV vaccination reduces severe respiratory infections in infants [17], and a group B streptococcus (GBS) vaccine has shown a favorable safety profile and successful antibody transfer [18]. Inactivated COVID-19 vaccines administered during pregnancy are safe for pregnant women and newborns [19].

This review demonstrated the safety and efficacy of vaccinating pregnant women against influenza, DTaP, RSV, GBS, and COVID-19. The key findings are as follows.

Influenza Vaccination

Influenza vaccines administered during pregnancy are safe and effective. Kostinov et al. found minimal local reactions and no systemic side effects, with protective antibody levels lasting up to nine months post-delivery [12]. Munoz et al. observed comparable safety profiles between pregnant and nonpregnant women, with efficient maternal antibody transfer resulting in higher cord blood antibody concentrations that decreased within six weeks postpartum [13]. Vesikari et al. demonstrated the immunogenicity of trivalent (IIV3) and quadrivalent (IIV4) inactivated vaccines, with IIV4 offering additional protection against the B/Yamagata strain [14]. Munoz et al. also verified the safety and strong immunogenicity of Fluzone® and Fluarix® vaccines [15]. These studies highlight the dual benefits of influenza vaccination in protecting mothers from complications during pregnancy and providing passive immunity to newborns.

DTaP Vaccination

Research has shown that administering the DTaP vaccine to pregnant women reduces newborn pertussis. Halperin et al. found that infants born to Tdap-vaccinated mothers had higher pertussis antibody levels than those born to unvaccinated mothers [16]. Despite a decrease in antibody concentrations during the infants’ initial immunization, particularly for certain antigens, early protection from maternal immunization is crucial in reducing the risk of pertussis in the first few months after birth.

RSV Vaccination

Research indicates that the RSV F-protein nanoparticle vaccine is safe and effective in pregnant women. Madhi et al. found that RSV-related MS-LRTI in infants increased by 39.4% within the first 90 days after birth and decreased severe RSV infections with hypoxemia by 58.8% [17]. These findings suggest that maternal immunization against RSV is a promising strategy for combating major respiratory illnesses in infants.

GBS Vaccination

Research has indicated that GBS vaccines are safe and effectively induce immune responses in pregnant women. Swamy et al. found that a single dose of a trivalent GBS vaccine administered between 24 and 34 weeks of pregnancy increased serotype-specific IgG concentrations 13- to 23-fold [18]. These antibodies were transmitted to newborns with placental transfer ratios of 0.62 to 0.82, and the elevated antibody levels in infants lasted for at least three months, offering essential early neonatal protection.

COVID-19 Vaccination

Research shows that COVID-19 vaccination during pregnancy is safe and effective, eliciting an immune response without significantly increasing adverse outcomes in mothers or newborns. Yang et al. found no significant differences between vaccinated and unvaccinated groups regarding premature membrane rupture, low birth weight, or NICU admission rates [19]. Although vaccinated mothers exhibited elevated serum alanine transaminase levels in the first trimester, these were not linked to adverse outcomes. In addition, maternal vaccination resulted in the transmission of SARS-CoV-2-specific antibodies to newborns, providing passive protection during their early and vulnerable months [20].

This review shows that vaccines administered to pregnant women elicit strong immune responses, efficiently transfer antibodies to newborns, and are safe for both mothers and babies in a consistent manner. Despite minor side effects, variations in antibody longevity among infants highlight the need for further studies to optimize vaccination timing and methods for enduring protection. These findings underscore the crucial role of immunizing expectant mothers to improve perinatal and neonatal health outcomes.

**Figure 2:**
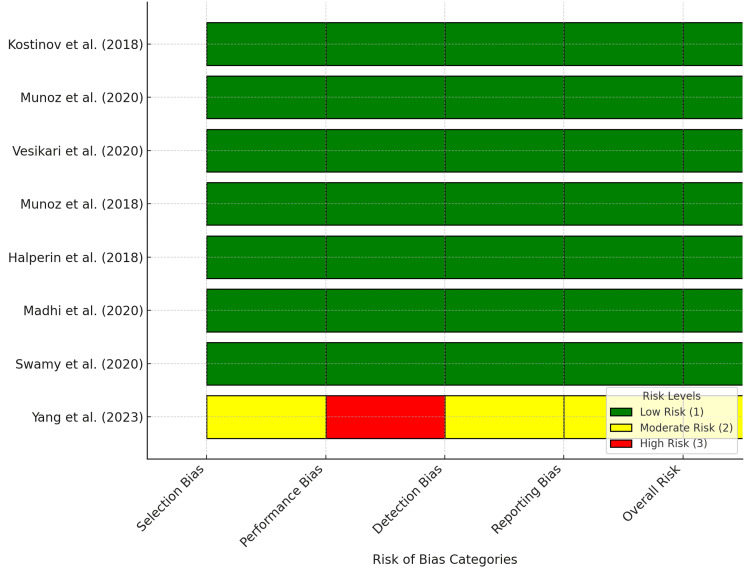
Individual risk of bias in systematic review studies on the safety and efficacy of vaccines during pregnancy The risk of bias for each study was assessed across categories including selection bias, performance bias, detection bias, reporting bias, and overall risk. Studies included in the analysis are as follows: Kostinov et al. (2018) [12], Munoz et al. (2020) [13], Vesikari et al. (2020) [14], Munoz et al. (2018) [15], Halperin et al. (2018) [16], Madhi et al. (2020) [17], Swamy et al. (2020) [18], and Yang et al. (2023) [19].

Eight studies, including seven RCTs and one observational study, were assessed for bias (Figure 2). The seven RCTs exhibited minimal bias across the selection, performance, detection, and reporting categories, affirming their methodological rigor and reliability [12-18]. Conversely, an observational study by Yang et al. showed moderate-to-high bias [19], moderate selection and detection bias, and significant performance bias due to challenges in blinding in a non-experimental context. The RCTs yielded robust evidence with negligible bias, whereas the observational studies provided useful but less rigorous data. These findings underscore the reliability of systematic reviews on vaccine safety and efficacy in pregnant women while acknowledging the limitations of non-randomized research.

Discussion

Evaluating vaccine safety and efficacy during pregnancy is crucial for maternal and infant health. This review highlights the safety of vaccines administered during pregnancy, with side effects similar to those in nonpregnant individuals (mostly mild to moderate), aligning with research confirming vaccine safety [4,21]. Studies on influenza vaccines across pregnancies have also shown no increase in the risk of adverse perinatal outcomes [7].

Mounting evidence demonstrates the effectiveness of COVID-19 vaccines in pregnant women, eliciting robust immune responses, high antibody levels, and significant infection prevention, which are all crucial for maternal and infant health [17]. Vaccinated pregnant women exhibit elevated antibody levels, providing essential protection against infections and related pregnancy complications, given the documented adverse effects of COVID-19 on pregnant women and perinatal outcomes. Vaccination markedly reduces COVID-19 infection rates in pregnant women, safeguards maternal health, and mitigates risks of preterm birth and severe maternal illness [22]. Maternal vaccination also confers passive immunity to infants through transplacental antibody transfer, thus offering early protection to newborns [23]. Studies indicate reduced SARS-CoV-2 infections among infants under 12 months born to vaccinated mothers, highlighting the benefits of maternal immunization for both mothers and infants, diminishing their risk of hospitalization and symptomatic illness [20,24].

These findings support the public health guidelines advocating vaccination during pregnancy to prevent infectious diseases associated with significant maternal and neonatal morbidity. Healthcare providers can reassure expectant patients using robust evidence, and effective vaccine communication is essential [25,26].

Influenza vaccines are safe for pregnant women, causing minimal side effects and not increasing adverse pregnancy outcomes [12,27]. These vaccines elicit robust maternal immune responses, produce protective antibodies that are transferred to the fetus, and provide neonatal protection [12,28]. Placental antibody transfer offers early defense against respiratory infections in newborns [15,17]. Maternal influenza vaccination significantly reduces infant influenza incidence and related complications through passive antibody protection [15,17]. Evidence supports the safety and immunogenicity of influenza vaccination during pregnancy, benefiting both mothers and infants. The findings emphasize the importance of including pregnant women in vaccination programs to enhance maternal and neonatal health while protecting two generations. Current evidence endorses the recommendation of influenza vaccination to pregnant women to prevent complications, highlighting it as a public health priority [28,29].

Research on DTaP vaccines has supported their safety and immunogenicity during pregnancy. Halperin et al. showed that Tdap vaccination during pregnancy induced robust immune responses in mothers and infants, providing early-life protection [16]. Tdap vaccines for expectant mothers are safe and effective, with studies indicating no significant increase in adverse pregnancy outcomes and minimal side effects [30-32]. Tdap immunization during pregnancy triggers robust immune responses, evidenced by increased antibody production crucial for protecting both mothers and infants against pertussis [30,33]. This dual protection enhances maternal health and offers passive immunity to newborns during their first months when they are most vulnerable to pertussis [33,34]. The evidence supports Tdap vaccination during pregnancy due to its excellent safety profile and ability to stimulate significant immune responses, making it critical in public health efforts to reduce pertussis incidence and improve maternal and infant health outcomes [28,30,31].

Madhi et al. confirm the safety of RSV vaccines during pregnancy, showing no increased risk of adverse outcomes [17,35], which agrees with research findings supporting inactivated vaccines in pregnancy [28]. RSV causes severe respiratory infections in newborns and often requires hospitalization. Maternal vaccination is essential for passive infant protection through transplacental antibody transfer, as demonstrated with Tdap and influenza vaccines [28,36]. Integrating RSV vaccines into maternal immunization programs can substantially reduce infant RSV infections and hospitalizations, potentially decreasing healthcare costs and improving neonatal health [37,38]. These findings support the safety and efficacy of RSV vaccines during pregnancy and advocate their inclusion in perinatal care to protect newborns from severe respiratory infections and enhance health outcomes [17,38].

Swamy et al.’s vaccine exhibited robust immunogenicity and safety in pregnant women, as confirmed by a randomized placebo-controlled phase II trial that showed strong GBS-specific antibody responses and no significant adverse effects [18,28]. Maternal GBS immunization safeguards infants through transplacental antibody transfer, crucial during the early months when newborns are most susceptible to GBS infection [18,39], akin to the Tdap vaccine’s protection against pertussis [28]. The GBS vaccine’s potential to safely and effectively prevent neonatal GBS infection suggests its significance in enhancing maternal vaccination strategies and protecting infants from severe GBS-related illnesses, supporting efforts to improve maternal and infant health through immunization [18,39].

Despite substantial evidence supporting vaccine safety and efficacy, vaccine hesitancy persists because of concerns regarding its long-term effects and a perceived lack of information on vaccination safety during pregnancy. Effective communication from healthcare providers is essential to address these concerns, as building trust and improving confidence in vaccination are crucial for increasing vaccine uptake among pregnant individuals.

This review has some limitations, including limited long-term infant health data post-maternal immunization, especially for newer vaccines such as COVID-19 mRNA vaccines, small sample sizes, and variations in study designs and vaccine types. Vaccine hesitancy persists because of concerns about the potential long-term effects and the lack of studies on the impact of targeted communication strategies on vaccination rates.

Most included RCTs were methodologically robust, yielding reliable outcomes on vaccine safety and effectiveness during pregnancy, with minimal selection, performance, detection, and reporting bias. Conversely, the observational study by Yang et al. showed a higher risk of bias due to non-randomized design constraints but provided valuable real-world evidence supporting COVID-19 vaccine safety in pregnancy, complementing RCTs [19]. This assessment emphasizes prioritizing high-quality RCTs while considering observational studies as supplementary evidence. Future research should focus on improving methodological rigor in non-randomized studies. The generally low risk of bias in the reviewed studies supports the conclusions of the systematic review, affirming vaccine safety and efficacy during pregnancy; however, the moderate to high risk in the observational study necessitates cautious interpretation. Continued research is crucial to support and build upon this evidence base.

Future research should focus on extensive longitudinal studies to evaluate the long-term health outcomes of babies of immunized mothers; investigate novel vaccine immunogenicity, efficacy, and safety during pregnancy in diverse multiethnic populations; assess public health messages and effectiveness of interventions in reducing vaccine hesitancy among pregnant individuals; and explore maternal antibody transfer mechanisms to optimize vaccination timing and methods for enhancing passive immunity in newborns.

## Conclusions

Maternal immunization is a safe and effective method for protecting mothers and infants from vaccine-preventable viral diseases such as influenza, pertussis, RSV, GBS, and COVID-19. RCTs have shown strong immune responses in pregnant women and successful transplacental antibody transfer, providing newborns with passive immunity. The low risk of bias in most RCTs supports their methodological quality, whereas the moderate-to-high bias in observational studies highlights challenges in non-randomized settings.

Despite strong evidence, vaccine hesitancy remains a significant treatment barrier, necessitating transparent evidence-based discussions by healthcare providers. Future studies should focus on long-term infant health outcomes, especially for new vaccines, and evaluate their safety and immunogenicity in diverse populations. Further research is required to optimize maternal immunization timing and enhance antibody persistence in neonates. Addressing vaccine hesitancy through tailored education and public health messaging is crucial. This review supports routine maternal vaccination programs, and further research is essential to address knowledge gaps and ensure equitable, informed access to immunization during pregnancy.
